# Chimeric antigen receptor-T-cell therapies going viral: latent and incidental viral infections

**DOI:** 10.1097/QCO.0000000000001066

**Published:** 2024-10-03

**Authors:** Eleftheria Kampouri, Gemma Reynolds, Benjamin W. Teh, Joshua A. Hill

**Affiliations:** a Infectious Diseases Service, Lausanne University Hospital and University of Lausanne, Lausanne, Switzerland; b Department of Infectious Diseases, Peter MacCallum Cancer Centre, Melbourne; c Sir Peter MacCallum Department of Oncology, University of Melbourne, Parkville; d National Centre for Infections in Cancer, Peter MacCallum Cancer Centre, Melbourne, Victoria, Australia; e Vaccine and Infectious Disease Division; f Clinical Research Division, Fred Hutchinson Cancer Center; g Department of Medicine, University of Washington, Seattle, Washington, USA

**Keywords:** chimeric antigen receptor-T, chimeric antigen receptor, cytomegalovirus, herpesviruses, respiratory, virus

## Abstract

**Purpose of review:**

Infections are the leading cause of non-relapse mortality following chimeric antigen receptor (CAR)-T-cell therapy, with viral infections being frequent both in the early and late phases post-infusion. We review the epidemiology of viral infections and discuss critical approaches to prevention and management strategies in this setting.

**Recent findings:**

Herpesviruses dominate the early period. herpes simplex virus and varicella zoster virus infections are rare due to widespread antiviral prophylaxis, but cytomegalovirus (CMV) reactivation is increasingly observed, particularly in high-risk groups including B cell maturation antigen (BCMA)-CAR-T-cell therapy recipients and patients receiving corticosteroids. While CMV end-organ disease is rare, CMV is associated with increased mortality, emphasizing the need to evaluate the broader impact of CMV on long-term hematological, infection, and survival outcomes. Human herpesvirus-6 (HHV-6) has also emerged as a concern, with its diagnosis complicated by overlapping symptoms with neurotoxicity, underscoring the importance of considering viral encephalitis in differential diagnoses. Respiratory viruses are the most common late infections with a higher incidence after BCMA CAR-T-cell therapy. Vaccination remains a critical preventive measure against respiratory viruses but may be less immunogenic following CAR-T-cell therapy. The optimal timing, type of vaccine, and dosing schedule require further investigation.

**Summary:**

A better understanding of viral epidemiology and preventive trials are needed to improve infection prevention practices and outcomes following CAR-T-cell therapies.

## INTRODUCTION

During the last decade, chimeric antigen receptor (CAR)-T-cell therapies targeting CD19 and B cell maturation antigen (BCMA) have revolutionized the management of multiple advanced hematologic malignancies. Several CD19-targeted products are currently approved for B-cell acute lymphoblastic leukemia [1,2], non-Hodgkin lymphomas [3–12], and chronic lymphocytic leukemia [13], and two BCMA-targeted products are available for multiple myeloma [14–17] (Table 1). Importantly, the place of these therapies is shifting from later to earlier, as soon as second line of treatment in non-Hodgkin lymphomas and multiple myeloma [7,8,18,19], and their use is generating exciting results in targeting solid tumors, autoimmune disease, and infection [20–23]. 

**Box 1:**
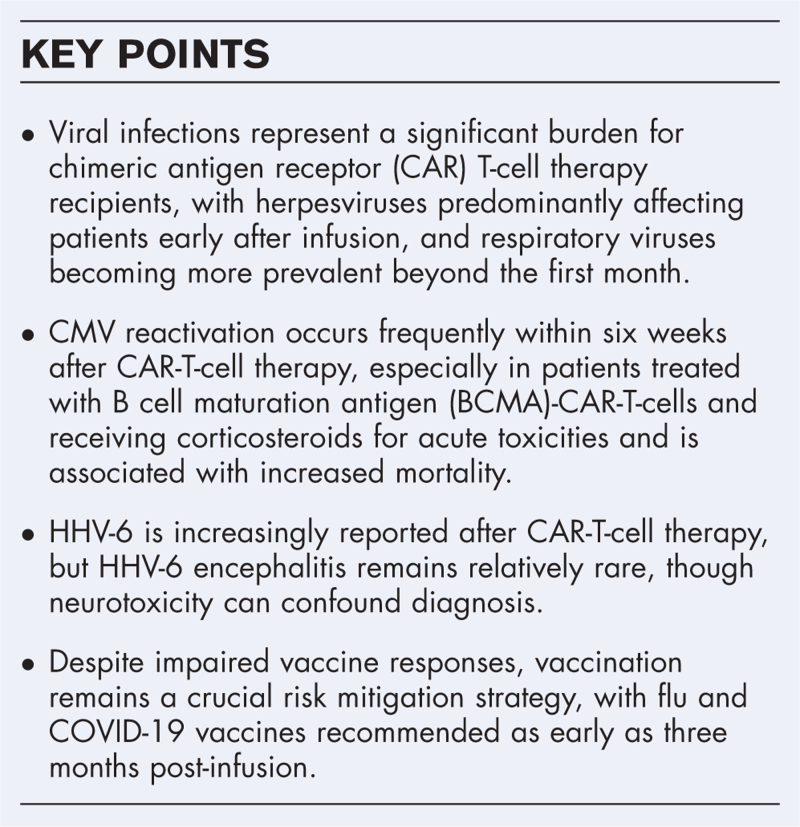
no caption available

**Table 1:** Approved CAR-T-cell products by indication

B-cell malignancy	CD19-targeted CAR-T-cell products
B-ALL	
<25 years-old	Tisagenlecleucel (Kymriah; Novartis)
>18 years-old	Brexucabtagene autoleucel (Tecartus; Kite/Gilead)
Large B-cell lymphoma	
After ≥2 lines of treatment	Tisagenlecleucel (Kymriah; Novartis)
After ≥1 line of treatment	Axicabtagene ciloleucel (Yescarta; Kite/Gilead)
After ≥1 line of treatment	Lisocabtagene maraleucel (Breyanzi; Juno/BMS)
Follicular lymphoma	
After ≥2 lines of treatment	Tisagenlecleucel (Kymriah; Novartis)
After ≥2 lines of treatment	Axicabtagene ciloleucel (Yescarta; Kite/Gilead)
After ≥1 line of treatment	Lisocabtagene maraleucel (Breyanzi; Juno/BMS)
Mantle-cell lymphoma	
After ≥2 lines of treatment	Brexucabtagene autoleucel (Tecartus; Kite/Gilead)
After ≥2 lines of treatment	Lisocabtagene maraleucel (Breyanzi; Juno/BMS)
CLL and SLL	
After ≥2 lines of treatment	Lisocabtagene maraleucel (Breyanzi; Juno/BMS)
Multiple Myeloma	BCMA-targeted CAR-T-cell products
After ≥2 lines of treatment	Idecabtagene vicleucel (Abecma; Celgene/BMS)
After ≥1 line of treatment	Ciltacabtegene autoleucel (Carvykti; Janssen/Legend)

B-ALL, B-cell acute lymphoblastic leukemia; BCMA, B cell maturation antigen; CAR, chimeric antigen receptor; CLL, chronic lymphocytic leukaemia; SLL, small lymphocytic lymphoma.

Despite their success, CAR-T-cell therapies are associated with unique and frequent immune-related toxicities including cytokine release syndrome (CRS), immune effector cell-associated neurotoxicity syndrome (ICANS), and less frequently secondary hemophagocytic lymphohistiocytosis (HLH)-like syndromes, often requiring immunosuppressive therapy such as corticosteroids and antiinterleukin agents [24–28]. Further, immune effector cell-associated hematological toxicity (ICAHT), including profound and prolonged neutropenia and “on-target off-tumor” effects leading to B-cell aplasia, represents the most common long-term adverse event with important clinical implications [29–31]. These factors, along with the high immunosuppressive burden of CAR-T-cell therapy recipients even before infusion related to the malignancy itself, prior lines of treatment, the lymphodepleting chemotherapies and bridging or concomitant therapies, all lead to high infection risk [32,33]. The temporal distribution of these risk factors shapes the timeline and epidemiology of infection [32]. Moreover, CD19 and BCMA-targeted products induce distinct humoral immunity deficits based on the expression of their targets on different stages of maturation of B-cells, impacting infection epidemiology and dictating nonidentical preventive approaches [31]. Bacterial and viral infections are the most frequent infections within the first month after infusion [32,34–37]. Herpesviruses including CMV and HHV-6 have recently been recognized as important pathogens occurring mainly during the first six weeks after infusion [38^▪^,39^▪^,40^▪^,41^▪^], while respiratory viruses are the most common late infections [34,42^▪▪^]. Conversely, invasive fungal infections remain relatively rare both in the early and late phases after CAR-T-cell therapy [32,43].

Substantial efforts on reporting, grading and management of immune toxicities have improved outcomes after CAR-T-cell therapy, yet infection prevention practices remain disproportionally unevolved and are largely based on expert opinion or extrapolated from the hematopoietic cell transplant (HCT) setting. Recent data revealed that while the frequent immune-related toxicities are responsible for a minority of non-relapse deaths, infections remain the single most important cause of non-relapse mortality, responsible for more than half of all non-relapse deaths, highlighting an urgent need for improvement of infection prevention practices [44^▪▪^,^45^^▪▪^]. Achieving better infection outcomes, and thereby reducing non-relapse mortality, will require a deeper understanding of infection epidemiology through prospective studies and enhanced standardized infection reporting [46]. This knowledge will guide the design of trials to assess preventive practices as we strive towards trial-generated evidence in lieu of expert opinion. Here we review risk factors for and the epidemiology of viral infections in CAR-T-cell therapy recipients and discuss critical approaches to prevention and management strategies with a special focus on viral monitoring and vaccination.

## HUMORAL AND CELLULAR IMMUNITY DEFICITS

### Humoral immunity following chimeric antigen receptor-T

Enduring B-cell aplasia is an expected “on-target, off-tumor” effect of CAR-T therapy targeting CD19 or BCMA antigens on B- and plasma cells. A major clinical consequence of B-cell aplasia is hypogammaglobulinemia. Baird *et al.* demonstrated that 48% of patients had not recovered immunoglobulin G (IgG) >400 g/dl by 1 year post infusion [47^▪▪^]. Despite this, pathogen-specific antibody levels are at least partially maintained following CD19-CAR-T-cell therapy. Seroprevalence studies examining antibodies against vaccine-preventable viral infections (VPVIs) in in CD19 CAR-T-cell therapy recipients have demonstrated high (75–100%) residual seropositivity against measles, mumps, rubella (MMR) and varicella zoster virus (VZV), in both the early period between 0 and 6 months postinfusion (74–96%) [48,49], as well as up to two years post treatment (58–92%) [50,51]. Lower rates of seroprotective titers have been observed for hepatitis viruses, and encapsulated bacteria including *S. pneumoniae* and *H. influenzae* type b [50,51], however baseline serological and vaccination status in these cohorts are unknown. Although data is scarce, retention of existing seropositivity may be significantly lower after BCMA-targeted CAR-T-cell therapies (0–75% seropositivity) compared to CD19 products [51,52]. This may potentially be due to absolute reduction in long-lived plasma-cells, responsible for maintaining pathogen-specific antibody titers, expressing BCMA but lacking CD19 from their surface [31,53,54]. The relationship between humoral deficiency, enduring seropositivity and incidence of viral infections has not been prospectively evaluated.

### Cellular immune deficits after chimeric antigen receptor-T

**FIGURE 1:**
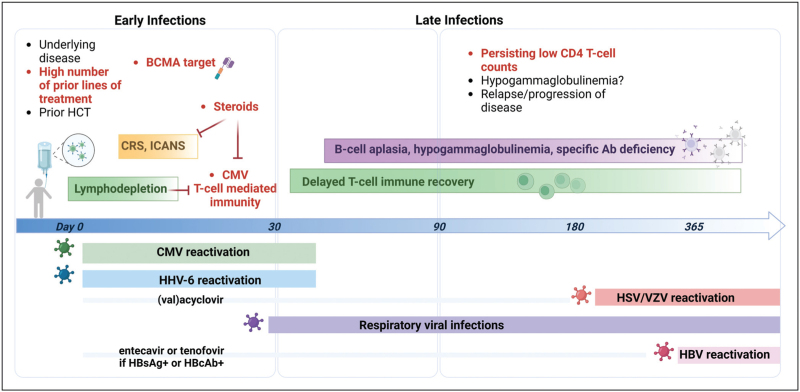
Risk factors and epidemiology of viral infection following CAR-T-cell therapy. Risk factors for viral infection are depicted in bullets along with the timeline of different viral infections divided into early (before 30 days) and late (after 30 days from infusion). Risk factors for viral infections identified in clinical studies are in red. CRS, cytokine release syndrome; HCT, hematopoietic cell transplant; ICANS, immune effector cell-associated neurotoxicity syndrome. Figure created with BioRender.com.

Cellular immunity following CAR-T-cell therapy is significantly impacted by lymphodepleting chemotherapy, corticosteroid administration for immune-mediated adverse events, and the lingering effects of prior hematological treatments. While data on the reconstitution of cellular immunity is less abundant than that for humoral immunity, existing studies indicate a delayed recovery of CD4^+^ and CD8^+^ effector T-cells following initial lymphodepletion. Despite variations in outcome measures, three key studies have evaluated CD4^+^ recovery at the 12-month mark. Baird *et al.*[47^▪▪^] reported that 60% of CAR-T-treated patients had CD4^+^ cell count below 200 cells/μl, whereas Locke *et al.*[55] found that 33% of the ZUMA-1 and ZUMA-9 cohort had not achieved normalized CD4^+^ cell count by 12 months. Logue *et al.* also observed that a subset of patients with baseline lymphopenia did not recover CD4^+^ cell count within 12 months, with a median count of 155 cells/μl [56^▪▪^]. The slow recovery of CD4^+^ cell count has important implications for infection risk, in particular reactivation of latent herpesviruses (Fig. 1).

## CYTOMEGALOVIRUS

### Incidence, viral kinetics and risk factors

The epidemiology and role of cytomegalovirus (CMV) in CAR-T-cell therapy recipients is not well elucidated due to scarcity of systematic data. CMV reactivation is reported in 40 to 60% of CMV seropositive patients within the first three months after CD19-CAR-T-cell infusion in retrospective studies with variable frequency of testing [40^▪^,^57^–^60^]. Data on BCMA CAR-T-cell therapies are scarcer [57,61]. In a prospective study among 72 CMV seropositive adults with weekly testing for up to 12 weeks, the cumulative incidence of CMV reactivation at any level was 27% by week 12 [38^▪^]. CMV reactivation mainly occurs between 2 and 6 weeks after CAR-T-cell infusion with median time to reactivation presenting little variation between studies (14–22 days postinfusion) [38^▪^,40^▪^,^58^–^60^]. Importantly, cumulative incidence CMV reactivation incidence was twice as high in BCMA- compared with CD19-CAR-T-cell therapy recipients (46% vs. 23%) and BCMA CAR-T-cell therapy was identified as a significant risk factor in multivariable analyses [38^▪^]. Importantly, BCMA CAR-T-cell therapy recipients often have a higher number of prior treatments, including prior HCT, which could account for this finding [38^▪^]. Of note, a higher number of prior lines of treatment was also potentially associated with increased risk for CMV [38^▪^].

Not surprisingly, the use of corticosteroids for CRS and/or ICANS has been identified as a strong predictor of CMV reactivation in multivariable analyses [38^▪^,39^▪^,40^▪^], particularly when administered for more than three days [38^▪^], and/or in combination with other immunosuppressive treatments (≥2 immunosuppressive drugs) [40^▪^]. CMV-specific T-cell mediated immunity (CMV-CMI) is key to controlling CMV replication, and its measurement can predict CMV risk in HCT recipients [62,63]. In one study, CMV-CMI was longitudinally evaluated in CAR-T-cell therapy recipients using an enzyme-linked immunospot assay (T-SPOT.CMV; Oxford Immunotec) to quantify T-cell responses to CMV antigens IE-1 and pp65 [38^▪^]. CMV-CMI values reached a nadir around week 2 and recovered to levels similar to prior to CAR-T-cell infusion around week 4, highlighting the period at highest risk for CMV [38^▪^]. Importantly, CMV-CMI was significantly impacted in patients receiving corticosteroids, indicating a mechanistic link between corticosteroid use and CMV reactivation [38^▪^]. Lower CMV-CMI at week 2 was associated with increased CMV risk, highlighting its utility in refining CMV risk assessment and identifying patients who could benefit from more stringent surveillance or prophylactic practices [38^▪^].

### Clinical significance

CMV viremia is rather frequent but the clinical significance and the risk of progression of CMV viremia to disease is largely unexplored [57,61]. CMV end-organ disease has been reported after CAR-T-cell therapy [59,64,65] but is infrequent [38^▪^,40^▪^,^58^,^60^,^66^]. However, a preemptive approach in which CMV treatment is administered above certain CMV detection thresholds was used in all cohort studies (therapy administered in 7%-71% of CMV events even in the absence of end-organ diseases), precluding an accurate assessment of the natural history of CMV reactivation [38^▪^,40^▪^,^57^–^60^,^66^]. A CMV syndrome manifested as fever, malaise, leukopenia, and/or thrombocytopenia, as described in solid organ transplant recipients, could be a prevalent manifestation in CAR-T-cell therapy recipients [67]. Indeed, criteria for CMV-syndrome diagnosis were present in 20% of all CMV events in one study, making this the primary clinical manifestation an important motif for CMV treatment initiation [40^▪^]. However, the frequent occurrence of fever and worsening cytopenias due to CAR-T-cell-related toxicities, and the absence of routine CMV monitoring, make the diagnosis of CMV syndrome challenging in this setting [57].

Finally, CMV is associated with significant “indirect effects” in HCT and solid organ transplant recipients. “Indirect effects” attributed to CMV include increased mortality [68–70], as well as a heightened risk for fungal and bacterial infections through immunomodulatory mechanisms [71]. Clinically significant CMV reactivation, especially early after infusion, is associated with a higher 1-year mortality [39^▪^,40^▪^], and a higher incidence of relapse/progression [40^▪^]. These findings highlight the need for evaluate the clinical impact of CMV in CAR-T-cell therapy recipients, beyond a binary vision of presence/absence of end-organ disease and incorporating long-term hematological, infectious and survival outcomes.

### Prevention considerations

Based on available data, CMV monitoring should be considered 2–6 weeks postinfusion for patients on corticosteroids for >3 days for CRS/ICANS, those receiving BCMA CAR-T-cell therapy, and those with extensive prior treatments [72]. However, the role of preemptive or prophylactic therapy for CMV have not been studied.

## HUMAN HERPESVIRUS-6

Several cases of human herpesvirus-6 (HHV-6) encephalitis following CAR-T-cell therapy have been reported, but the epidemiology and clinical significance of HHV-6 reactivation in this context remain largely unknown [73]. HHV-6 encephalitis can be clinically indistinguishable from neurological symptoms due to ICANS, which occurs in over 70% of CAR-T-cell therapy patients [74,75]. In the absence of routine surveillance, the incidence of HHV-6 reactivation and encephalitis may be underestimated. Furthermore, HHV-6 is associated with various other manifestations beyond encephalitis, such as fever, pneumonia, and delayed immune reconstitution, which could be overlooked or misattributed to other causes following CAR-T-cell therapy [73].

In a prospective cohort of 89 CD19 and BCMA CAR-T-cell therapy recipients with weekly testing, the cumulative incidence of HHV-6 reactivation was 6% within 12 weeks from infusion [41^▪^]. Reactivation occurred within 2 and 6 weeks postinfusion and was mainly low level and self-limiting. In a larger retrospective cohort of >600 CAR-T-cell therapy recipients with targeted symptom-driven sampling, only one case of possible HHV-6 encephalitis was diagnosed, though the low rate of plasma and cerebrospinal fluid testing was a main limitation [41^▪^]. While reassuring, these data do not definitely address all questions pertaining to the role and impact of HHV-6 reactivation, underscoring the need to remain vigilant, especially in the setting of novel cellular therapies such as allogeneic CAR-T-cell products and new biologics for the management of acute immune toxicities [76]. Nonetheless, in light of existing data, routine HHV-6 monitoring does not seem warranted, but HHV-6 testing should be performed in blood and CSF in patients with compatible clinical manifestations [72].

## HERPES SIMPLEX VIRUS AND VARICELLA-ZOSTER VIRUS

Specific data on the incidence of herpes simplex virus (HSV) and varicella-zoster virus (VZV) infections following CAR-T-cell therapy are lacking. Despite a high rate of VZV reactivation when prophylaxis is not used, the reactivation of HSV or VZV has become rare due to the widespread use of (val)acyclovir prophylaxis during the first few months after infusion [77,78]. Breakthrough infections on prophylaxis are rarely reported [64,79], including a case of fatal HSV pneumonia [64]. Late reactivation following prophylaxis cessation are more frequently reported and could reflect the delayed immune recovery of CD4^+^ T-cells [35,80–83].

Routine antiviral prophylaxis with acyclovir or valacyclovir for HSV and VZV in seropositive patients is recommended and should be started at lymphodepleting chemotherapy. The optimal duration is not well defined, but a minimum of 6 months is often recommended and a CD4^+^ cell count above 200 cells/mm^3^ could be used as a surrogate to stop prophylaxis [32,72]. Finally, given the ongoing risk for herpes zoster, vaccination using the recombinant herpes zoster vaccination (Shingrix) is recommended approximately 6–12 months after CAR-T-cell therapy when acyclovir prophylaxis is discontinued [72].

## EPSTEIN–BARR VIRUS

The incidence and clinical significance of EBV viremia following CAR-T-cell therapy remain unclear. Sporadic cases of EBV detection have been reported after CD19 (*n* = 1) and BCMA CAR-T-cell therapy (*n* = 4), all of which were asymptomatic and did not require anti-EBV intervention [34,82].

Importantly, EBV is associated with posttransplant lymphoproliferative disorders (PTLD) in patients with profound immunosuppression, such as those who have undergone solid organ transplants or HCT. EBV-mediated PTLD has been reported after CAR-T-cell therapy, including a case of lethal T-cell lymphoma [84,85]. Notably, these cases have occurred in patients treated with CAR-T-cells for EBV-positive B-cell malignancies. These findings highlight the importance of ongoing vigilance in monitoring for secondary cancers following CAR-T-cell therapy [86]. EBV monitoring following CAR-T-cell therapy is not warranted in the absence of clinical suspicion of infection or PTLD [72].

## HEPATITIS VIRUSES AND HUMAN IMMUNODEFICIENCY VIRUS

Outcomes for over 275 patients with chronic or resolved hepatitis B (HBV) infections after CAR-T therapy are summarized in Table S1, Supplemental Digital Content. Antiviral prophylaxis was consistently provided to those with chronic infections, while approaches varied for resolved infections [87–89]. Patients with resolved infections (HBsAg−/HBcAb+) were most often given prophylaxis, representing an approximate prophylaxis rate of 30% (Table S1, Supplemental Digital Content) [90,91]. Data on prophylaxis duration was limited. In the aggregated cohort (*n* = 275) (Table S1, Supplemental Digital Content), 17 (6%) reactivations were reported (Table S1, Supplemental Digital Content), including four cases of reactivation in patients with resolved HBV, and an additional 3 HBV reactivations identified from case reports [92,93]. In patients with chronic infection on antiviral therapy, increases in HBV viral load occurred in patients co-expressing HBeAg, indicating high infectivity (*n* = 3) [94,95]. In patients with resolved infections, reactivation occurred in those lacking HBsAb or when prophylaxis was discontinued early (typically < 3 months) [95,96]. In a cohort without prophylaxis, 2 out of 30 patients experienced reactivation within 14 months [97]. The incidence of acute hepatitis accompanying HBV reactivation was low [88,91,98], although rises in alanine transaminase were occasionally observed [87,96].

Patients with chronic hepatitis C or HIV were excluded from clinical trials, so outcomes after cellular therapies are not well documented. A case report of one patient with hepatitis C showed no increase in HCV viral load or hepatitis after CAR-T therapy [96]. A recent review identified six patients living with HIV treated with CAR-T for lymphoma, with clinical outcomes similar to those of patients without HIV infection [99].

### Prevention

Patients undergoing either CD19 or BCMA CAR-T-cell therapy with a history of both chronic (HBsAg+) and resolved HBV (HBsAg−/HBcAb+) should receive antiviral prophylaxis with entecavir or tenofovir for at least 12 months after CAR-T-cell therapy [72,100,101]. If antiviral prophylaxis is not used, monthly monitoring of HBV viral load and liver enzymes with preemptive HBV therapy upon detection of reactivation is critical [72].

## RESPIRATORY VIRUSES

Our understanding of the real-world incidence of respiratory viral infections (RVIs) in CAR-T treated patients is evolving. In patients with non-Hodgkin lymphoma, the estimated incidence of RVIs likely lies between 0.6 and 1.4 events per 100 patient years (calculated from Table S2, Supplemental Digital Content), with a higher incidence observed in patients with multiple myeloma [102,103]. However, most published data capture RVIs incidence during the SARS-CoV-2 pandemic, where some centers reported a significant reduction in the number of RVIs due to globally enhanced infection prevention measures [104^▪▪^]. Thus the incidence of RVIs among CAR-T-cell therapy recipients may increase post-pandemic.

RVIs are typically considered a late infectious complication of CAR-T therapy, with higher prevalence after day 30 [34,35,42^▪▪^,^105^]. The proportion of total infection events caused by RVIs increases with follow-up duration; RVIs contributed a higher proportion of viral infections in studies reporting infections between 6 and 12 months (4–44%) [35,79,106,107], compared with 3-months or less (7–26%) [34,66]. In a summary of sentinel trials, excluding SARS-CoV-2 infection, rhinovirus was the most common respiratory virus (34% of RVIs) followed by influenza (22%) and parainfluenza (15%) [108^▪▪^]. Single-center observational studies have reported higher rates of RSV (18–27% of RVIs) [103,109].

However, data on the severity of non-SARS-CoV-2 RVIs are sparce. Fatal influenza was reported in low numbers in both the pivotal trials (*n* = 1) [55], and subsequent observational studies (*n* = 2) [110], but the proportion of non-SARS-CoV-2 RVIs requiring hospital admissions is unclear. The severity of SARS-CoV-2 infection in patients with hematological malignancies, including CAR-T-cell therapy, has been reviewed extensively elsewhere [111–114]. Longitudinal data from the EBMT registry, reflecting predominately patients with non-Hodgkin lymphoma, demonstrates a reduction between 2020 and 2022 in SARS-CoV-2 mortality (44 vs. 8%), mechanical ventilation (44 vs. 11%) and hospital admission (92 vs. 43%), mirroring increasing uptake of vaccination and antiviral therapies [115].

### Vaccination for RVIs

Post-vaccine seroconversion studies have primarily examined response to SARS-CoV-2 vaccines in patients receiving CD19-targeted CAR-T-cell therapy. An early systematic review, evaluating response rates to mRNA vaccines reported a 38% response rate (*n* = 70) to a single dose [116], with a recent meta-analysis showing only a 17% of patients who remained seronegative after two doses would convert after the third dose [117]. Recent data from a prospective, longitudinal cohort study found that in a population of CAR-T-cell therapy recipients previously unvaccinated for SARS-CoV-2, 4 or more vaccines may improve humoral immunogenicity, but median spike IgG titers achieved relative to healthy controls was not reported [118]. Early data suggests patients treated with a BCMA-targeted CAR-T-cell therapy have a higher rate of seroconversion to SARS-CoV-2 vaccine, between 76 and 80% after two doses [118,119]. Regarding influenza vaccination, a prospective study of BCMA and CD19 CAR-T-cell therapy recipients vaccinated with quadrivalent influenza at a median of 20 months postinfusion, demonstrated that 31–40% had robust (4-fold) increase in at least one vaccine target [120^▪^]. A higher rate of seroconversion to influenza vaccine was again observed following treated with BMCA CAR-T compared to CD19 product [120^▪^]. Importantly, T-cell specific responses are generated in most CAR-T patients to SARS-CoV-2 vaccines [121–125], and influenza [126], even in the absence of neutralizing antibodies [48,121]. To date, response to the recombinant RSV vaccine has not been studied in CAR-T-cell therapy recipients.

Defining the optimal timing of re-vaccination after CAR-T is an area of ongoing research. Two studies have prospectively evaluated vaccine response to viral vaccines by timing of administration [120^▪^,^121^]. Walti *et al.* evaluated the humoral response to an inactivated influenza vaccine administered pre- or post-CAR-T-cell therapy, demonstrating robust antibody production prior to CAR-T-cell therapy with a subsequent decline by day 90–120 after CAR-T-cell therapy [120^▪^]. Nonetheless, it demonstrates the ability to establish immunity that bridges high-risk periods of immune suppression. Hill *et al.* extended these findings by demonstrating that precellular therapy vaccination was associated with increased antibody titers following post-infusion vaccination [121]. Furthermore, this longitudinal study evaluated the response to SARS-CoV-2 vaccination at either <4 months, or 4–12 months, after CAR-T-cell therapy [121]. Fewer CAR-T patients demonstrated neutralizing antibody titers (58%) than either allogeneic (70%) or autologous (69%) transplant recipients, but antibody titers did not differ significantly based on time of initiation post-CAR-T-cell therapy [121]. Taken together, these results suggest that vaccination pre-CAR-T-cell therapy, combined with post-CAR-T-cell therapy vaccination, may optimize vaccine immunogenicity in CAR-T-cell therapy recipients. Future studies will need to prospectively evaluate this approach.

### Predictors of vaccine response

Several studies have evaluated predictors of vaccine responses against viral infection, again predominately in SARS-CoV-2 vaccines in patients with non-Hodgkin lymphoma. Significant predictors of improved vaccine response include vaccination or SARS-CoV-2 infection prior to cellular therapy [121,122], and higher circulating B-cell counts [121,127,128]. It should be noted that in broader cohorts, patients with non-Hodgkin lymphoma [123,129], and patients treated with CAR-T-cells [123,129–131], have demonstrated less robust vaccine responses compared to patients with other hematological malignancies or treatments. Limited or no association has been identified between patient age, sex, absolute lymphocyte count, or time since CAR-T-cell therapy [120^▪^,^121^,^132^]. These factors should be prospectively evaluated in studies examining individualized approaches to vaccination of CAR-T-cell therapy recipients [133^▪^].

## CONCLUSIONS AND FUTURE DIRECTIONS

Despite recent advancements in our understanding of infection epidemiology, significant gaps remain. The frequent reactivation of latent viruses poses unanswered questions about their clinical impact, their role in hematological outcomes, and their potential implications for immune-related toxicities. To address these uncertainties, large, ideally prospective, studies with long-term follow-up are needed. The findings from these studies would be essential for informing best practices in infection prevention, which should then be validated through controlled clinical trials. The recent COVID-19 pandemic has highlighted critical aspects of vaccinating CAR-T-cell therapy recipients, but further research is required to determine the optimal timing for vaccine initiation, as well as the most effective vaccine type, dosage, and schedule. Finally, enhanced diagnostics and omics-based approaches could offer deeper insights into the viral landscape in cellular therapy recipients, shedding light on clinical syndromes at the intersection of immune toxicities and infection.

## Acknowledgements

*None.*

*Author contributions: E.K. and G.R. conceptualized, co-wrote the manuscript, and created the figure and tables. B.W.T. and J.A.H. co-wrote and edited the manuscript.*

### Financial support and sponsorship

*None.*

### Conflicts of interest

*E.K. has received grants from the Swiss National Science Foundation (SNSF grant n P500PM_202961) and SICPA Foundation.*

*G.R. receives research funding from NHMRC PhD Scholarship #2013970 and speaker fees paid to institution from Janssen.*

*B.W.T. has received grants from MSD, Seqirus, Sanofi all paid to institution; honoraria – from Alexxion, Pfizer, Gilead all paid to institution; Advisory − CSL-Behring, Takeda, Moderna all paid to institution.*

*J.A.H. has served as a consultant for Moderna, Allovir, Gilead, Karius, Geovax, CSL Behring, and received research funding from Allovir, Takeda, Geovax, and Merck. All other authors do not report any conflicts of interest.*

## Supplementary Material

Supplemental Digital Content

## Data Availability

*The data that support the findings of this study are available from the corresponding author upon reasonable request.*

## REFERENCES AND RECOMMENDED READING

Papers of particular interest, published within the annual period of review, have been highlighted as:

▪ of special interest
▪▪ of outstanding interest
