# Supplementary material for: Chimeric antigen receptor-T-cell therapies going viral: latent and incidental viral infections
Source: Curr Opin Infect Dis. 2024 Oct 3;37(6):526–35. doi: 10.1097/QCO.0000000000001066 (PMC11932447; doi:10.1097/QCO.0000000000001066)
Supplement: Supplemental Digital Content [file coidi-37-526-s001.docx]

**Supplement to** **CAR-T-cell Therapies Going Viral: Latent and Incidental Viral Infections**

**Table S1**: Outcomes in patients with chronic or resolved HBV infection after CAR-T-cell therapy

| **Study, year** | **N** | **Treatment/**  **HM** | **Serostatus** | **Prophylactic**  **strategy** | **Agent** | **Duration** | **Monitoring** | **Reactivation** |
| --- | --- | --- | --- | --- | --- | --- | --- | --- |
| Strati, 2019^1^ | 2 | CD19/  NHL | HBsAg (1/2)  HBcAb (2/2)  HBsAb (2/2) | Universal | ETV | >12 months | PCR HBV | 1/2;  after stop of prophylaxis |
| Han,  2020^2^ | 9 | BCMA/  MM | HBsAg (1/9)  HBcAb (9/9)  HBsAb (6/9) | Chronic infection (HBsAg+) | ETV or 3TC | NR | PCR HBV | 0/9;  median FUP 9.8 months |
| Wang,  2020^3^ | 41 | Mixed population | HBsAg (12/41)  HBcAb (41/41)  HBsAb (41/41) | Chronic infection (HBsAg+) | ETV, 3TC or TDF | NR | PCR HBV | 3/41;  1 without prophylaxis |
| Cao,  2020^4^ | 56 | CD19 and CD22/  ALL, NHL | HBsAg (19/56)  HBcAb (37/56)  HBsAb (51/56) | Risk stratified  (HBsAg+ or HBsAb-) | ETV | 15.6 months (IQR: 6.3–22.2) | PCR HBV | 1/56;  4 months post-infusion |
| Yang,  2020^5^ | 15 | CD19/  R/R DLBCL | HBsAg (15/15)  HBeAg (3/15)  HBcAb (14/15) | Universal | 3TC, ETV, or TDF | 12 months | PCR HBV | 3/15;  1 month (2 patients) and 5 months (1 patient) post-infusion |
| Lai,  2020^6^ | 3 | CD19/  ALL | HBsAg (3/3)  HBsAb (0/3)  HBcAb (2/3) | Universal (but all were HBsAg+) | ETV or TDF | NR | PCR HBV | 0/3 |
| Fu,  2023^7^ | 50 | BCMA/  MM | HBsAg (7/50)  HBcAb (50/50) | Chronic infection  (HBsAg+) | ETV or 3TC | >12 months | PCR HBV | 4/50;  3 with resolved infection |
| Cui,  2021^8^ | 20 | CD19/  NHL, ALL | HBsAg (5/20)  HBeAg (3/20)  HBcAb (20/20) | Risk stratified  (HBsAg+ or HBsAb-) | ETV or 3TC | Individualized | PCR HBV | 1/20;  6 months post-infusion (4 months post-HCT), after stop of prophylaxis |
| Kong,  2023^9^ | 31 | CD19/  DLBCL | HBsAg (6/31)  HBsAb (13/31)  HBcAb (17/31) | Risk stratified  (HBsAg+) | ETV or ETV + ADV | Individualized; until last follow-up | PCR HBV | 2/31;  1 with chronic and 1 with resolved infection |
| Li,  2021^10^ | 30 | CD19/  NHL, ALL | HBsAg (0/30)  HBsAb (21/30)  HBcAb (30/30) | No prophylaxis | NA | N/A | PCR HBV | 2/30;  2 and 14 months post-infusion |
| Liu,  2019^11^ | 17 | CD19/  NHL, ALL | HBsAg (6/17)  HBcAb (17/17) | Risk stratified  (HBsAg+) | ETV | Individualized | PCR HBV | 0/17 |

ETV: entecavir, 3TC: lamivudine, TDF: Tenofovir disoproxil, ADV: Adefovir dipivoxil, ALL: acute lymphoblastic leukemia, FUP: follow-up, HCT: hematopoietic cell transplantation, HM: hematologic malignancies, IQR: interquartile range, NA: non applicable, NHL: non-Hodgkin lymphoma, NR: not reported, R/R DLBCL: relapsed/refractory diffuse large B-cell lymphoma.

**Table S2**: Summary of the proportion of all-grade infections represented by respiratory viral infections in clinical trials and cohort studies.

| **Study, year** | **Disease** | **CAR-T-cell product** | **N** | **Total number of any-grade infection events*** | **RVIs (%)**** | **Median follow-up (days)** |
| --- | --- | --- | --- | --- | --- | --- |
| Park 2018^12^ | ALL | CD19 | 53 | 41 | 11 (27) | 180 |
| Zeng 2020^13^ | NHL | CD19/CD22 | 14 | 3 | 0 (0) | 321 |
| Locke 2021^14^ | NHL | CD19 | 108 | 104 | 13 (13) | 810 |
| Gaut 2021^15^ | NHL | CD19 | 22 | 5 | 0 (0) | 30 |
| Thakkar 2021^16^ | NHL | CD19 | 19 | 25 | 9 (36) | 730° |
| Baird 2021^17^ | NHL | CD19 | 41 | 73 | 14 (19)ǂ | 582 |
| Beyar-Katz 2022^18^ | NHL | CD19 | 60 | 32 | 3 (9) | 30 |
| Neill 2020^19^ | NHL | CD19 | 60 | 63 | 15 (24) | NR |
| Wudhikarn 2020^20^ | NHL | CD19 | 60 | 101 | 27 (27)^ǂ^ | 365 |
| Logue 2021^21^ | NHL | CD19 | 85 | 70 | 29 (41) | 384 |
| Wong 2020^22^ | NHL | CD19 | 31 | 18 | 8 (44) | 180 |
| Rejeski 2022^23^ | NHL | CD19 | 248 | 163 | 13 (8) | 90 |
| Kambhampati 2022^24^ | MM | Anti-BCMA | 55 | 47 | 25 (53) | 180 |
| Zhu 2021^25^ | NHL: 34  ALL: 58 | Anti-CD19 | 92^α^ | 24 | 3 (13) | 180 |
| Hill 2018^26^ | NHL:62 ALL:47 | Anti-CD19 | 133 | 66 | 20 (30) | 90 |

ALL: acute lymphoblastic leukemia; NHL: non-Hodgkin lymphoma; RVI: respiratory viral infections

*Any-grade microbiologically confirmed or clinically-defined infection as reported by the referenced manuscript or abstract.

**Percentage of total any-grade infection events represented by respiratory virus infections.

°Infections beyond day 30.

^ǂ^Includes clinically-defined infections thought viral in etiology (n =4).

^α^Infections evaluated in a cohort of 92 patients (of 113 patients treated).

**References**

1. Strati P, Nastoupil LJ, Fayad LE, Samaniego F, Adkins S, Neelapu SS. Safety of CAR T-cell therapy in patients with B-cell lymphoma and chronic hepatitis B or C virus infection. *Blood, The Journal of the American Society of Hematology*. 2019;133(26):2800-2802.

2. Han L, Zhou J, Zhou K, et al. Safety and efficacy of CAR-T cell targeting BCMA in patients with multiple myeloma coinfected with chronic hepatitis B virus. *J Immunother Cancer*. 2020;8(2).

3. Wang Y, Liu Y, Tan X, et al. Safety and efficacy of chimeric antigen receptor (CAR)-T-cell therapy in persons  with advanced B-cell cancers and hepatitis B virus-infection. *Leukemia*. 2020;34(10):2704-2707. doi:10.1038/s41375-020-0936-4

4. Cao W, Wei J, Wang N, et al. Entecavir prophylaxis for hepatitis B virus reactivation in patients with CAR T-cell therapy. *Blood, The Journal of the American Society of Hematology*. 2020;136(4):516-519.

5. Yang C, Xie M, Zhang K, et al. Risk of HBV reactivation post CD19-CAR-T cell therapy in DLBCL patients with concomitant chronic HBV infection. *Leukemia*. 2020;34(11):3055-3059.

6. Lai P, Chen X, Qin L, et al. The efficacy and safety of CAR-T cell therapy in patients with refractory ALL and concomitant HBV infection. *Leukemia*. 2020;34(10):2790-2793.

7. Fu S, Zhang Q, Jing R, et al. HBV reactivation in patients with chronic or resolved HBV infection following BCMA-targeted CAR-T cell therapy. *Bone Marrow Transplant*. 2023;58(6):701-709.

8. Cui R, Lyu C, Li Q, et al. Humanized anti‐CD19 chimeric antigen receptor‐T cell therapy is safe and effective in lymphoma and leukemia patients with chronic and resolved hepatitis B virus infection. *Hematol Oncol*. 2021;39(1):75-86.

9. Kong D, Ping N, Gao X, et al. Efficacy and safety of chimeric antigen receptor T cell therapy in relapsed/refractory diffuse large B-cell lymphoma with different HBV status: a retrospective study from a single center. *Front Immunol*. 2023;14:1200748.

10. Li P, Zhou L, Ye S, et al. Risk of HBV reactivation in patients with resolved HBV infection receiving anti-CD19 chimeric antigen receptor T cell therapy without antiviral prophylaxis. *Front Immunol*. 2021;12:638678.

11. Li P, Zhou L, Ye S, et al. Risk of HBV Reactivation in Patients With Resolved HBV Infection Receiving Anti-CD19 Chimeric Antigen Receptor T Cell Therapy Without Antiviral Prophylaxis. *Front Immunol*. 2021;12. doi:10.3389/FIMMU.2021.638678

12. Park JH, Romero FA, Taur Y, et al. Cytokine release syndrome grade as a predictive marker for infections in patients with relapsed or refractory b-cell acute lymphoblastic leukemia treated with chimeric antigen receptor T cells. *Clinical Infectious Diseases*. 2018;67(4):533-540. doi:10.1093/cid/ciy152

13. Zeng C, Cheng J, Li T, et al. Efficacy and toxicity for CD22/CD19 chimeric antigen receptor T-cell therapy in patients with relapsed/refractory aggressive B-cell lymphoma involving the gastrointestinal tract. *Cytotherapy*. 2020;22(3):166-171. doi:10.1016/j.jcyt.2020.01.008

14. Locke FL, Miklos DB, Jacobson CA, et al. Axicabtagene Ciloleucel as Second-Line Therapy for Large B-Cell Lymphoma. *New England Journal of Medicine*. 2021;386(7):640-654. doi:10.1056/NEJMoa2116133

15. Gaut D, Tang K, Sim MS, Duong T, Young P, Sasine J. Filgrastim associations with CAR T-cell therapy. *Int J Cancer*. 2021;148(5):1192-1196. doi:http://dx.doi.org/10.1002/ijc.33356

16. Thakkar A, Cui Z, Peeke SZ, et al. Patterns of leukocyte recovery predict infectious complications after CD19 CAR-T cell therapy in a real-world setting. *Stem Cell Investig*. 2021;8:18. doi:https://dx.doi.org/10.21037/sci-2021-008

17. Baird JH, Epstein DJ, Tamaresis JS, et al. Immune reconstitution and infectious complications following axicabtagene ciloleucel therapy for large B-cell lymphoma. *Blood Adv*. 2021;5(1):143-155. doi:http://dx.doi.org/10.1182/bloodadvances.2020002732

18. Beyar-Katz O, Kikozashvili N, Bar On Y, et al. Characteristics and recognition of early infections in patients treated with commercial anti-CD19 CAR-T cells. *Eur J Haematol*. 2021;108(July):1-9. doi:10.1111/ejh.13712

19. Neill L, Mackenzie SC, Marzolini MA V, et al. Steroid use, advanced stage disease and >=3 lines of prior chemotherapy are associated with a higher risk of infection following CD19 CAR T-cell therapy for B-NHL: Real world data from a large UK center. *Blood*. 2020;136(SUPPL 1):20-21. doi:http://dx.doi.org/10.1182/blood-2020-138865

20. Wudhikarn K, Pennisi M, Recio MG, et al. Infectious Complications in Aggressive B Cell Non-Hodgkin Lymphoma after CD-19 Chimeric Antigen Receptor T Cell Therapy. *Biology of Blood and Marrow Transplantation*. 2020;26(3 Supplement):S326. doi:http://dx.doi.org/10.1016/j.bbmt.2019.12.350

21. Logue JM, Zucchetti E, Bachmeier CA, et al. Immune reconstitution and associated infections following axicabtagene ciloleucel in relapsed or refractory large B-cell lymphoma. *Haematologica*. 2021;106(4):978-986. doi:http://dx.doi.org/10.3324/haematol.2019.238634

22. Wong SM, Shah M, Inam S, Kuhnl A, Sanderson R. Real-world data from Kings College Hospital: Infection complications post CAR-T treatment in high-grade B cell non-hodgkin lymphoma. *Bone Marrow Transplant*. 2020;55((Wong, Shah, Inam, Kuhnl, Sanderson) Kings College Hospital NHS Foundation Trust, London, United Kingdom):237. doi:https://dx.doi.org/10.1038/s41409-020-01120-w

23. Rejeski K, Perez A, Iacoboni G, et al. The CAR-HEMATOTOX risk-stratifies patients for severe infections and disease progression after CD19 CAR-T in R/R LBCL. *J Immunother Cancer*. 2022;10(5):e004475. doi:10.1136/jitc-2021-004475

24. Kambhampati S, Sheng Y, Huang CY, et al. Infectious complications in relapsed refractory multiple myeloma patients after BCMA Car t-cell therapy. *Blood Adv*. Published online September 2021. doi:10.1182/bloodadvances.2020004079

25. Zhu F, Wei G, Liu Y, et al. Incidence and Risk Factors Associated with Infection after Chimeric Antigen Receptor T Cell Therapy for Relapsed/Refractory B-cell Malignancies. *Cell Transplant*. 2021;30(79):1-9. doi:10.1177/09636897211025503

26. Hill JA, Li D, Hay KA, et al. Infectious complications of CD19-targeted chimeric antigen receptor-modified T-cell immunotherapy. *Blood*. 2018;131(1):121-130. doi:10.1182/blood-2017-07-793760
